# Hypertension and Diabetes in Akatsi South District, Ghana: Modeling and Forecasting

**DOI:** 10.1155/2022/9690964

**Published:** 2022-02-09

**Authors:** Dorothy O. Asante, Anita N. Walker, Theodora A. Seidu, Senam A. Kpogo, Jianjun Zou

**Affiliations:** ^1^School of Basic Medicine and Clinical Pharmacy, China Pharmaceutical University, Nanjing 211198, China; ^2^Department of Clinical Pharmacology, Nanjing First Hospital, Nanjing Medical University, Nanjing 210006, China; ^3^School of Public Health, Nanjing Medical University, Nanjing 211166, China; ^4^School of Pharmacy, Department of Pharmaceutics, China Pharmaceutical University, Nanjing 211198, China; ^5^School of Nursing and Midwifery, University of Health and Allied Sciences, PMB 31, Ghana; ^6^Department Pharmacy, Nanjing First Hospital, China Pharmaceutical University, Nanjing 210006, China

## Abstract

**Background:**

The rising incidence of hypertension and diabetes calls for a global response. Hypertension and diabetes will rise in Ghana as the population ages, urbanization increases, and people lead unhealthy lives. Our goal was to create a time series algorithm that effectively predicts future increases to help preventative medicine and health care intervention strategies by preparing health care practitioners to control health problems.

**Methods:**

Data on hypertension and diabetes from January 2016 to December 2020 were obtained from three health facilities. To detect patterns and predict data from a particular time series, three forecasting algorithms (SARIMAX (seasonal autoregressive integrated moving average with exogenous components), ARIMA (autoregressive integrated moving average), and LSTM (long short-term memory networks)) were implemented. We assessed the model's ability to perform by calculating the root mean square error (RMSE), mean absolute error (MAE), mean square error (MSE), and mean absolute percentage error (MAPE).

**Results:**

The RMSE, MSE, MAE, and MAPE for ARIMA (5, 2, 4), SARIMAX (1, 1,  1) × (1, 1, 1,  7), and LSTM was 28, 769.02, 22, and 7%, 67, 4473, 56, and 14%, and 36, 1307, 27, and 8.6%, respectively. We chose ARIMA (5, 2, 4) as a more suitable model due to its lower error metrics when compared to the others.

**Conclusion:**

All models had promising predictability and predicted a rise in the number of cases in the future, and this was essential for administrative and management planning. For appropriate and efficient strategic planning and control, the prognosis was useful enough than would have been possible without it.

## 1. Introduction

More often than not, hypertension (HT) and diabetes mellitus (DM) coexist and have become major worldwide health issues with a significant impact on cardiovascular morbidity and mortality [1]. Globally, an estimated 1.13 billion and 463 million people (i.e., 1 in every 11 adults (20-79 years) are affected with HT and DM, respectively [2, 3]. In addition to draining household resources and increasing healthcare expenses, they also put the World Health Organization's (WHO) goal of decreasing 1/3 of morbidity and mortality rates from noncommunicable diseases (NCDs) by 2030 in jeopardy [4, 5]. Therefore, finding models that accurately predict future increases in HT and DM is critical for tailoring prevention treatments and streamlining intervention programs.

People in economically developing nations are more susceptible to suffer from hypertension and diabetes [6], and Ghana is no exception like any of these countries. In Ghana, they are the leading cause of noncommunicable diseases (NCDs) [7]. HT is the third leading cause of hospitalizations and deaths accounting for 4.7 percent of the total hospitalizations and 15.3 percent of the total fatalities [8]. Additionally, for the past 15 years, it has ranked among the top five most prevalent outpatient diseases [9]. It continues to be the most important predictor of stroke in Ghana, with a population-attributable risk of approximately 91 percent [10]. Diabetes, on the other hand, was reported to have a prevalence of 9.26 percent in some urban areas in Ghana in 2009. However, it had a 2 percent prevalence in 1964 when Apollonius first identified it. According to a survey published by the Ministry of Health (MOH) in 2012, hypertension and diabetes affect 19-48 percent and up to 9 percent of Ghanaian adults, correspondingly [9]. Nevertheless, current research shows that Ghanaians have hypertension and diabetes prevalence rates of 30.3% and 6.46 %, respectively [11, 12].

For assessing health or illness status and predicting future events, time series can be invaluable. A mathematical model can be extrapolated since it uses the progression of historical datasets across time. However, given the complexity of the underlying techniques, their use remains largely unexplored [13]. Using time series to model clinical data has become increasingly popular [14, 15]. However, there is a paucity of research utilizing a time series approach to study the incidence of DM and HT in individuals. Those that do exist are mostly concerned with the occurrence of DM or HT in the urban areas [16, 17]. HT affects the majority of diabetic patients [1]; also, there is a rising trend in HT and DM among Ghanaian rural communities [18, 19]. Therefore, understanding this trend effectively minimizes inpatient care and improves clinical outcomes.

To our knowledge, no research has used time series and LSTM models to investigate the prevalence of patients with coexisting HT and DM, or DM and HT, in a rural Ghanaian community/district. To effectively plan and regulate the development of HT and DM in individuals in Akatsi South, we created three forecasting algorithms to identify patterns and forecast the growth of these conditions in individuals.

## 2. Materials and Methods

### 2.1. Study Area

**Figure 1:**
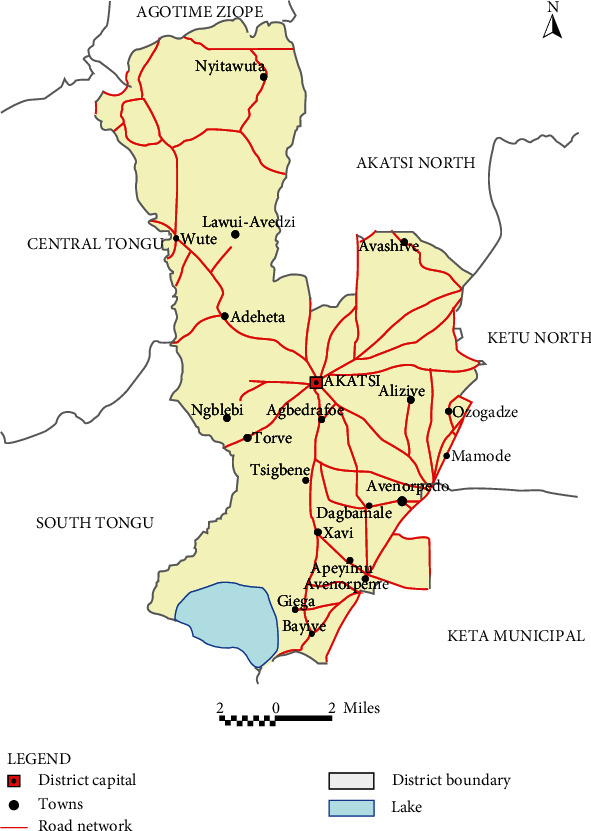
Map of Akatsi South District. The map of Akatsi South in the district context showing the various district capitals and towns.

Akatsi South District (see Figure 1) is predominantly rural, with rural towns comprising 2/3 of the district's population (67.7 percent). At the same time, the district's urban population accounts for 32.3% of total residents. It has a total of 29 health centres throughout the area. HT is among the top 10 diseases in the district [20, 21]. Since HT and DM are comorbidities [1], it is necessary to investigate the general trend of HT and DM in individuals in order to integrate the forecasting model effectively into the existing disease control program and thereby decrease the rate of associated health problems.

### 2.2. Data Collection

We gathered the data from Akatsi District Hospital, Sepe Clinic, and Wute Health Centre in the district. We received 2178 patients with coexisting HT and DM data from the hospital folders at various outpatient departments. Study participants were eligible if they had a medical history of diabetes, had high blood pressure, or had been previously diagnosed as hypertensive by any health care professional. During the study period, individuals of all ages who received prehospital care from any facility were classified as having “hyperglycemia” or “hypoglycemia.” For evaluating hypertension, we used the 2017 American College of Cardiology/American Heart Association High Blood Pressure Guideline [22]. Hypertension is defined as a blood pressure reading of 140/90 mmHg or greater. The hypertension and blood glucose levels recorded in patients' medical charts during routine check-ups were considered response variables in the study. Other independent/predictor variables considered were age (years), weight (kg), smoking and drinking status, diet and physical exercise, sex, and family history of hypertension and/or diabetes.

We divided the data into two categories: training and testing. Training data is data from 2016 to 2019 (36 months). Data from 2020, which is 12 months long, is used to see how effective the models are at predicting the future. All facilities provided monthly data with no missing months. We uploaded the “*SampleDate*” column in a numeric format “*yyyymm*” in order to generate a time series in year/month, allowing us to decompose the data by month. Two additional columns were also created. Thus, the “total column” to indicate the total number of cases (comm1 + comm2 + comm3) and the “month_name” were later used in visualizations. comm1, comm2, and comm3 represent data from Akatsi District Hospital, Sepe Clinic, and Wute Health Centre, respectively. Anonymized and de-identified data were used to protect the privacy of patients, health care professionals, and the hospital in this study.

### 2.3. Statistical Data Analysis

Python 3.6 and TensorFlow 2.0 were used to code and analyze the data. Python is a well-known high-level programming language for general-purpose application. The intent behind designing this language was code readability. Additionally, some essential libraries in Python to implement all the work are Pandas, Statsmodels, Matplotlib, and Seaborn. A total of three forecasting methods were used in the study.

### 2.4. The ARIMA and SARIMAX model

The autoregressive integrated moving average (ARIMA) modeling procedure was carried out using monthly data from the three selected facilities during the study period. The autocorrelation function (ACF) and partial autocorrelation function (PACF) plots in time series analysis can be used to observe the stationary condition of a data series, and the augmented Dickey–Fuller (ADF) test statistic can be used to verify it. In addition, to determine the order of seasonality, the ACF and PACF plots can be used. When the ACF and PACF components display spikes following differencing, it indicates that a seasonal component can be included. Likewise, when time series data is nonstationary, the augmented Dickey–Fuller (ADF) [23] unit root (to obtain a *p* value of 0.05 or less) is useful to make the data stationary, allowing forecasting using the ARIMA model, or nonstationarity (*p* value > 0.05) is maintained, and the seasonal ARIMA with exogenous factor (SARIMAX) modeling can be used.

Models such as ARIMA use previous observations to predict future values by using lag parameter values, asserting that the trend will continue to hold. It merges autoregression (AR) and moving average (MA) models, as well as a sequence's differencing preprocessing phase to make it stationary, which is the integration step (I).

ARIMA is constructed by combining AR, I, and MA. It is also known as a nonseasonal ARIMA model. In order to accomplish it, it uses the differencing of raw observations. AR uses a mathematical technique such as linear regression to anticipate the next data point by studying the data points that have come before it [24]. The time series is made stationary by subtracting an observation from an observation from a prior time step. MA, on the other hand, makes use of the link between an observation and residual errors from a lagged moving average model. By substituting integer values for the model's parameters, ARIMA uses a standard notation known as *p*, *d*, and *q* to express a specific ARIMA model. The nonseasonal ARIMA model in this example can be stated as follows:

(1) $$\begin{array}{c} y_{t}^{\prime }=\text{c}+\phi_{1}y_{t-1}^{\prime }+\cdots +\phi_{p}y_{t-p}^{\prime }+\theta_{1}\varepsilon_{t-1}+\cdots +\theta_{q}\varepsilon_{t-q}+\varepsilon_{t}, \end{array}$$

where the differenced series is represented by *y*_*t*_′. Lagged values of *y*_*t*_ (*ϕ*_1_*y*_*t*−1_′ + ⋯+*ϕ*_*p*_*y*_*t*−*p*_′) and lagged errors (*θ*_1_*ε*_*t*−1_+⋯+*θ*_*q*_*ε*_*t*−*q*_) are represented on the right by the “predictors” column. In this case, the ARIMA (*p*, *d*, and *q*) model is used, with the following parameters: *p* (AR) denotes the number of lag observations, also known as the lag order; *d* (I) denotes the number of times the raw observations are differentiated, also known as the degree of differencing; and *q* (MA) denotes the size of the moving average term, also known as the order of moving average or lagged errors. When components are combined to form complex models, backshift notations (*B*) are employed. For example, the nonseasonal ARIMA model can be written in terms of *B* as follows:

(2) $$\begin{array}{c} \left(1-\phi_{1}B-\cdots -\phi_{1}\;B^{p}\right)\;{\left(1-B\right)}^{d}y_{t}=c+\left(1+\theta_{1}B+\cdots +\theta_{q}\;B^{q}\right)\varepsilon_{t}, \end{array}$$

where (1 − *ϕ*_1_*B*−⋯−*ϕ*_1_ *B*^*p*^) = *AR*(*p*), (1 − *B*)^*d*^*y*_*t*_ = *differences* (*d*), and *MA* (*q*) = (1 + *θ*_1_*B*+⋯+*θ*_*q*_ *B*^*q*^)*ε*_*t*_.

SARIMAX requires four additional orders, the first three of which are seasonal forms of the ARIMA orders. The fourth or final orders represent the length of the cycle. In this case, the parameters of the SARIMAX model are *p*, *d*, and *q* (*P*; *D*; *Q*; and *S*), for which *p* denotes nonseasonal autoregressive (AR) order, *d* denotes nonseasonal differencing, *q* denotes nonseasonal moving average (MA) order, *P* denotes seasonal AR order, *D* denotes seasonal differencing, *Q* denotes seasonal moving average (MA) order, and *S* denotes the length of repeating seasonal pattern. The following is the general equation:

(3) $$\begin{array}{c} \phi_{p}\left(L\right){\tilde{\phi }}_{P}\left(L^{s}\right){∆}^{d}{∆}_{s}^{D}y_{t}=\theta_{q}\left(L\right){\tilde{\phi }}_{Q}\left(L^{s}\right){∆}^{d}{∆}_{s}^{D}y_{t}\varepsilon_{t}+\overset{\Pi }{\underset{i=1}{\sum }}B_{i}x_{t}^{i}, \end{array}$$

where *ϕ*_*p*_(*L*) and  *θ*_*q*_(*L*) are nonseasonal AR and MA lag polynomials, ${\tilde{\phi }}_{P}\left(L^{s}\right)$ and ${\tilde{\phi }}_{Q}\left(L^{s}\right)$ are seasonal AR and MA lag polynomials, respectively, the time series, differenced *d* times, and seasonally differenced *D* times are ∆^*d*^∆_*s*_^*D*^*y*_*t*_, and ∑_*i*=1_^*Π*^*B*_*i*_*x*_*t*_^*i*^ is the exogenous term.

**Figure 2:**
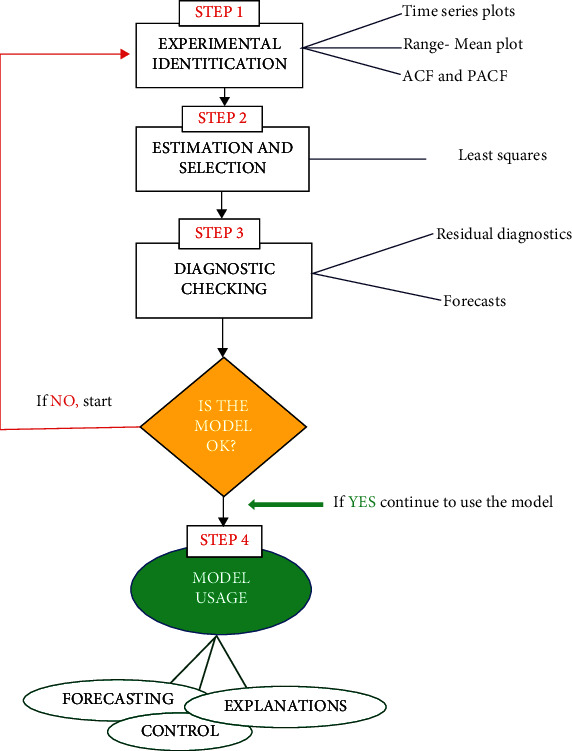
Box-Jenkins approach. A summary of the Box-Jenkins approach used in the implementation of our study.

As shown in Figure 2, models were fitted using the Box-Jenkins method [25]. Monthly case counts have shown an increasing trend over the year. An analysis of the time series yielded *Y*_*t*_ = *S*_*t*_ + *T*_*t*_ + *E*_*t*_, where *Y*_*t*_, *S*_*t*_, *T*_*t*_, and *E*_*t*_ are the actual data plot, seasonal component, the trend (which was differenced to achieve seasonality), and the residual component. Based on the Akaike information criteria (AIC), the best SARIMA and ARMA parameters were determined. The AIC uses the probabilistic method for estimating the range of ARMA models. The logarithm of $\left({\tilde{o}}^{2}\left(k\right)\right)+\left(2/T\right)\left(k\right)$ is thus the AIC. Both the autocorrelation function (ACF) and partial autocorrelation function (PACF) were plotted to test stationarity and lags and determine the order of MA and AR terms within each model. There were many ways to model the AR and MA terms. Statistical Science Research relies heavily on the accurate use of error metrics. Model efficiency is skewed if you choose an incorrect error metric. Therefore, the overall accuracy metrics are calculated and defined as follows:

MAE (mean absolute error) was determined as the average of the prediction error values, in which all the prediction values obtained are required to be positive to obtain the mean absolute error. It is given by

(4) $$\begin{array}{c} \text{MAE}=\frac{1}{a}\overset{a}{\underset{r=1}{\sum }}\left(W_{r}-{\hat{W}}_{r}\right). \end{array}$$

MAPE (mean absolute percentage error) was determined as the average absolute percent error for each period minus actual values divided by real values and often expressed as a percentage given by the formula:

(5) $$\begin{array}{c} \text{MAPE}=\overset{a}{\underset{r=1}{\sum }}\left(\frac{W_{r}-{\hat{W}}_{r}}{W_{r}}\right)\times \frac{100}{a}. \end{array}$$

The RMSE (root mean square error) model performance can be assessed in the near term by periodically comparing the actual difference between a predicted and reported value. At times, it can be expressed as a percentage given by the formula:

(6) $$\begin{array}{c} \text{RMSE}=\sqrt{\frac{\sum_{r=1}^{a}{\left(w_{r}-{\overset{\wedge }{w}}_{r}\right)}^{2}}{a}}, \end{array}$$

(7) $$\begin{array}{c} \text{RMSE}\left(\%\right)=\frac{\sqrt{\sum_{r=1}^{a}{\left(w_{r}-{\overset{\wedge }{w}}_{r}\right)}^{2}}}{1/a\sum_{r=1}^{a}{\hat{w}}_{r}}\times 100. \end{array}$$

To determine the mean squared error (or MSE), the squared forecast error numbers are averaged together and divided by two. In order to make the forecast errors positive, squaring them increases the weight given to significant errors. The formula is

(8) $$\begin{array}{c} \text{MSE}=\frac{1}{a}\overset{a}{\underset{r}{\sum }}{\left(W_{r}-{\overset{\wedge }{W}}_{r}\right)}^{2}. \end{array}$$

The lower the prediction error, the better the model. For all formulas, *W*_*r*_ is the actual number of cases for month “*r*,” ${\hat{w}}_{r}$ is the expected number of cases for month “*r*,” and *a* is the total observations.

### 2.5. The Long Short-Term Memory (LSTM) Model

Recurrent neural networks (RNN) identify trends in sequential data and are used in data mining applications including forecasting. In a traditional neural network, all of the inputs are treated as if they were completely separate from one another. An issue with this method is that it cannot be used for tasks that require the network to recall events from previous data. Hochreiter and Schmidhuber developed LSTM networks, which are a type of RNN, to prevent the long-term dependency limitation [26]. It is possible to use LSTM to create clinical decision support systems to manage coexisting hypertension and diabetes patients because the cells of the LSTM network's memory blocks allow it to store long-term data and control the amount of data kept in the entire network. Thus, the LSTM manages to keep, forget, or ignore data points based on a probabilistic model by employing a series of “gates,” each with its own RNN.

**Figure 3:**
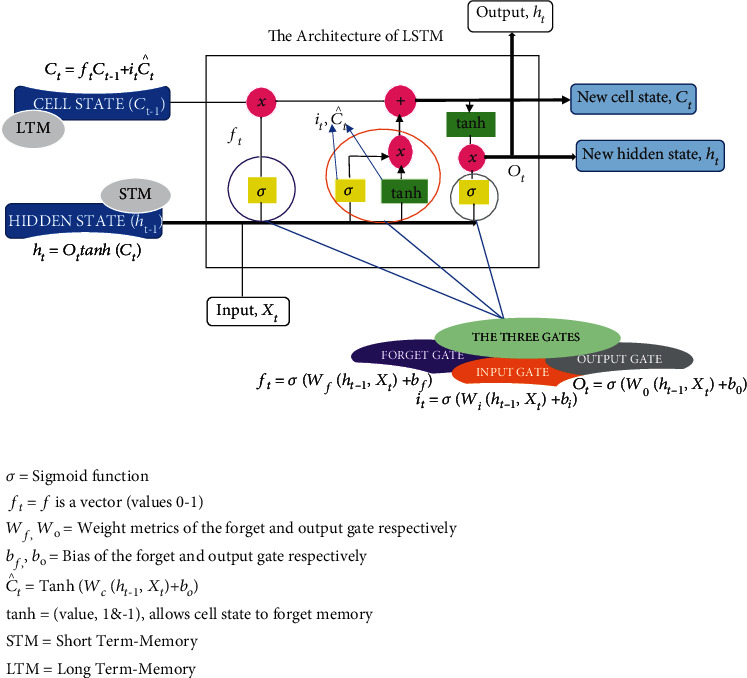
LSTM architecture. The structure of LSTM, showing the gates and their equations.

Connected in every possible way, the three gates (forget, input, and output) in LSTM make it highly effective at handling temporal correlation for time series data.  Figure 3 depicts the structure of an LSTM, with the various gates and equations [27]. Aside from that, LSTMs have two distinct states between the cells. This refers to the cell and hidden states responsible for storing long- and short-term memories, respectively. The cell state serves as a conveyor belt to ensure that information flows in the same direction throughout the entire network, from start to finish, whereas the “forget gate” is a critical element that acts as a transition between different time steps within the hidden layer, ensuring that the cell state is controlled and accurate throughout the entire process. The LSTM algorithm is well-suited for classifying, processing, and forecasting time series when time lags of unknown duration are present.

**Figure 4:**
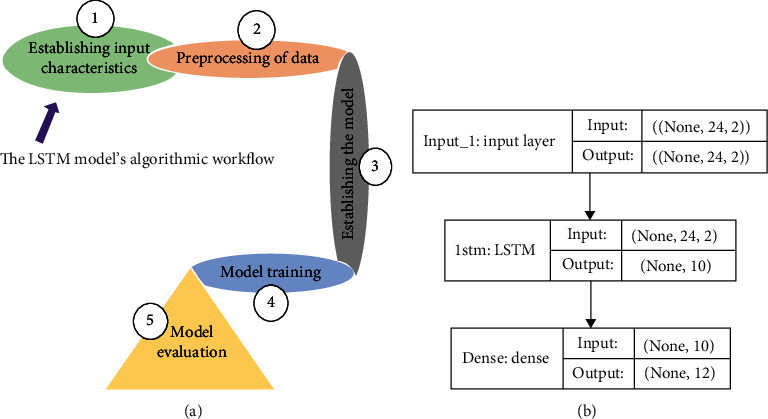
(a) Process flow of the LSTM model used for this study; (b) Architecture of the LSTM model generated in the study.

Here, we used a simple LSTM using Python and the TensorFlow framework. TensorFlow is a machine learning framework developed by Google that is free and open source. We installed the TensorFlow module using Python. Then the libraries were to define input-output data, allocate test and training data sets, build, fit a model, optimize the model for batch and epoch sizes, train the mode, and finally validate the accuracy scores for prediction output. The data for LSTM is prepared and preprocessed in a manner that differs significantly from that of the other algorithms in several ways. Before we begin preprocessing the data, few parameters were established first. They are first; the number of previous timestamps to use for predictions was indicated by the term “lag,” and second is lookahead—this parameter specified the number of timestamps in the future that we needed to predict. Then, we implemented a simple LSTM architecture with lag = 24 and lookahead = 12. Thus, we used 2 years of previous data to predict 1 year into the future. After, we used a 90/10 ratio for the training set and testing set. With an Early Stopping callback that stops training when the model's validation loss no longer decreases, the training process is accelerated after the initialization of 1000 epochs for the training process.  Figure 4 shows the process flow used and the architecture of our LSTM model. Using the results obtained, we selected the best model based on the test set's lowest RMSE, MSE, MAE, and MAPE.

## 3. Results

**Figure 5:**
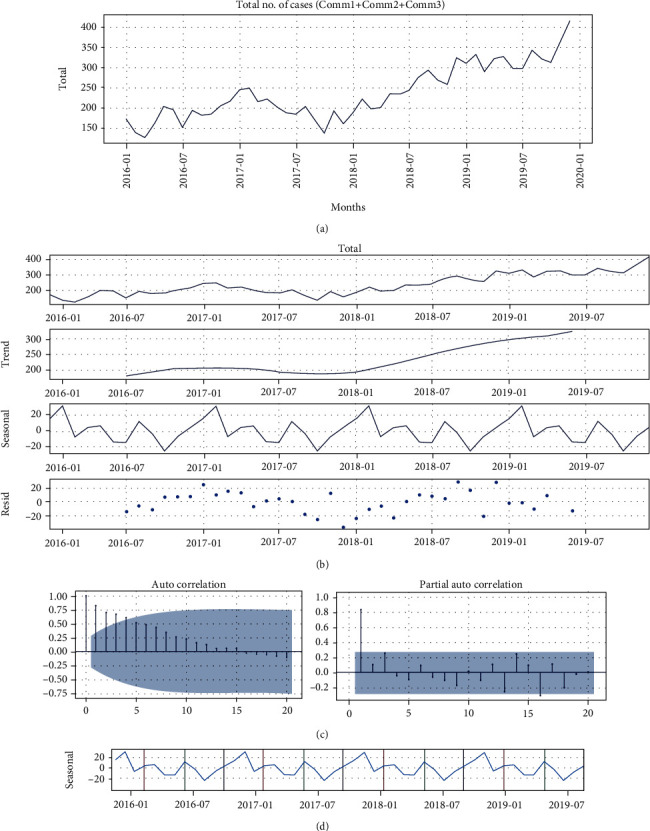
(a) The overall trend of the number of monthly cases; (b) The time series decomposition graph showing the seasonal trend; (c) Autocorrelation and Partial Autocorrelation plots of HT and DM cases; (d) Seasonality graph indicated with different color bands.

The annual prevalence of hypertension and diabetes in patients differed significantly from year to year (see Figure 5(a)), with a mean of 60 cases per year. In December 2019 and March 2016, the greatest and smallest percentage deviations were recorded, respectively. *Y*_*t*_ = *S*_*t*_ + *T*_*t*_ + *E*_*t*_ was the decomposition of the time series (see Figure 5(b)), where *Y*_*t*_, *S*_*t*_, *T*_*t*_, and *E*_*t*_ represent the actual data plot, seasonality, trend, and residual component, respectively. To achieve stationarity, the trend of the data points had to be differentiated from the actual data plot, and the seasonality of the data points had to be taken into account. The residual part of the data was also taken into account. Using the ADF test, the log-returns for the series were nonstationary (*p* = 0.2463) and needed differencing before they could be considered stationary. You can capture patterns that repeat themselves throughout the year using the seasonal component.

We created Figure 5(d) with different color bands to better understand the seasonality within the data. The graph shows that the pattern repeats itself every seven months, remaining the same in the following years. We applied a partial autocorrelation (see Figure 5(c)) to evaluate the AR model's order (*p*) as the autocorrelation steadily decreased. First, we require a stationary time series to use the ARIMA model for forecasting. Therefore, the use of second-order differentiation (*d* = 2) was conducted to avoid predictive imbalance in the time series under consideration. After being subjected to *d* = 2, it met the ADF test for time series data (*p* = 0.031). To narrow down the number of potential ARIMA models for further model selection, the AIC values for three models were compared because they all met the condition of white noise for the residual time series (see Table 1).

From Table 1, though ARIMA models (5, 2, 1) and (5, 2, 2) recorded the lowest AIC values, respectively, in the fitting process of testing, they however exhibited poor performances (RMSE = 37 and 43; MSE = 789.67 and 799.75; and MAE = 31 and 39) with the highest confidence interval of the predicted value vs. the actual value (MAPE = 11%and 19%), respectively. Given this, we selected ARIMA (5, 2, 4) as a suitable model due to its better performance during testing (RMSE = 28; MSE = 769.02; and MAE = 22) with the lowest confidence interval of the predicted value vs. the actual value (MAPE = 7%). However, it had the highest AIC value (AIC = 338.30). Second, the ideal ARIMA (*p*, *d*, *q*) (*P*, *D*, *Q*, *S*) time series data model parameters were determined programmatically by a Python script written in Python. The “grid search” (also known as hyperparameter optimization) was used to explore various combinations of the parameter continuously. We created a new seasonal ARIMA model with the SARIMAX function from the Statsmodels module for each combination of parameters and evaluated the overall quality of the model for each combination. To narrow down the number of potential SARIMAX models for further model selection, the AIC values for three models were compared because they all met the condition of white noise for the residual time series (see Table 2).

The plot_diagnostics function was used to construct our model diagnostics which ensured our model's residuals were nonstationary, were properly distributed, and had a skewness of 0. SARIMAX (1, 1,  1) × (1, 1, 1,  7) was considered a suitable model due to its better performance during testing (RMSE = 67; MSE = 4473; and MAE = 56) with the lowest confidence interval of the predicted value vs. the actual value (MAPE = 14%) as well as the lowest AIC value (AIC = 282.50).

Third, the long short-term memory model was built in 3 parts: (1) in the initial stage, the collected information was classified into two groups: the preceding two years' data were used as the testing sample, while the remaining data were used as the training dataset. When building a model and finding new potential relationships in data, the training samples were used, and the test samples were used to assess the model's performance built from the training dataset. (2) Then, using *X* values as time steps, a set of LSTM models were built. Assuming the time step was sixty, the sixty-first data was forecasted using the last 60 sets of data as input. The ideal model had the lowest RMSE, and (3) at last, the incidence was forecasted using the optimum model with the least RMSE allocation. We normalized our data using the MinMaxScaler; the adaptive moment estimation (Adam) optimizer and the RMSE were used as our model validation metrics. We further calculated for the MSE, MAPE, and MAE to enable better model selection (see Table 3).

**Figure 6:**
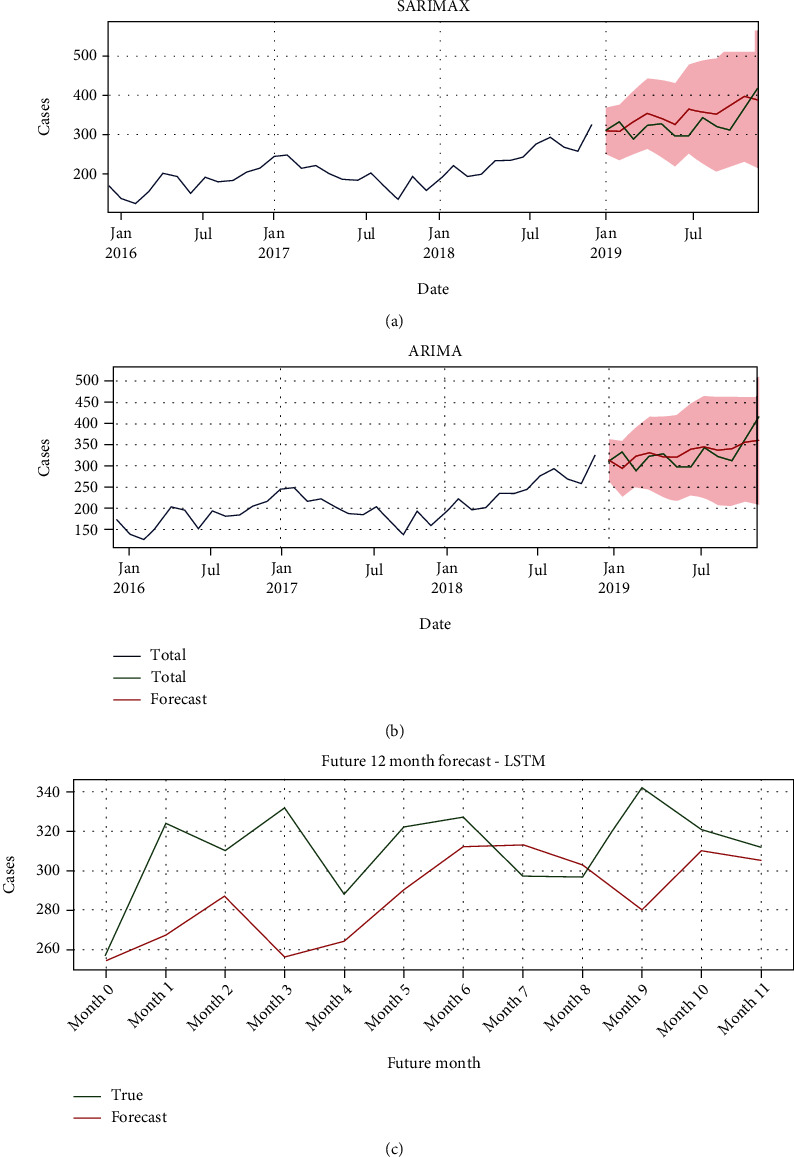
(a) Forecast of the SARIMAX (1, 1,  1) × (1, 1, 1,  7) model. (b) Forecast of the ARIMA (5, 2, 4) model. (c) Forecast of the LSTM model.

We selected the best three performing models (see Table 4), and based on that, we plotted (see Figure 6) the real and forecasted values of the HT and DM cases time series to evaluate our performance for each of the selected model and to finally enable selection of the best performing model in terms of forecasting. For the LSTM, we took a random sample of 12 continuous observations from the test set and the 12 predictions to draw the chart for result comparison.

From Figure 6, all three selected models estimated the number of cases to increase gradually with time which is accurate compared to the actual number of cases. Therefore, since our goal is to find a forecast that minimizes the errors, the ARIMA model with a lag value of 5 = AR was chosen as the best forecasting model due to its low errors. It utilizes I = 2 to make the time series stationary and MA = 4 for forecasting as shown in Table 4.

## 4. Discussion

Studying the temporal trends of individuals with coexisting hypertension and diabetes in a Ghanaian community, we projected potential incidence in order to aid in the prevention, treatment, and management of high blood pressure-high blood glucose diagnoses. As long as there are no adequate and efficient intervention mechanisms, the cases will continue to occur and may increase soon. Comparing our results, all three forecasting models considered for the study performed well indicating that monthly incidence of coexisting HT/DM patients in Akatsi South may be estimated using these models. The ARIMA model outperformed the SARIMAX and LSTM models in predicting the number of patients in the future. However, comparing our model's performance is difficult due to the absence of published health forecasts on our study topic using LSTM and the variety of modeling approaches and evaluation methods. To be clear, it was not a rigorous evaluation of several forecasting methods, but rather a choice of the method that best fits our data.

Applying time series modeling to forecast future occurrences is becoming more common in health care. ARIMA, SARIMAX, and LSTM can analyze and predict time series data. The ARIMA, ARIMAX, SARIMA, and SARIMAX time series prediction models have unique advantages over other methods. These models have been used in a variety of studies [17, 28, 29]. A possible explanation for the increasing trend in the survey, which had a 7-month seasonality, could be attributed in part to the recent climate change in the district, which is accompanied by heavy rains during the major rainfall period between July and September [20]. In addition, we presume that poor diet and lack of exercise are typical during the rainy, cold, and wet seasons, which is likely to increase the risk of obesity, cardiovascular disease, and diabetes.

Furthermore, the ARIMA model outperformed the LSTM because LSTM is more advanced, recognizing data sequence, like nonlinearities and complexities produce better results for long-term modeling and situations where dataset is large. In addition, regardless of extensive parameter tuning, an LSTM network trained on one dataset is likely to perform poorly on another. In short, the pretty small dataset and simplistic LSTM architecture may explain why the LSTM model underperformed compared to the ARIMA model. Nonetheless, since the LSTM also performed better than the SARIMAX, it could suggest that utilizing a more complex LSTM architecture with more data may improve outcomes.

There are, of course, some drawbacks to our research. First, the incidence of hypertension and diabetes may be underestimated due to an individual's lack of awareness of their blood pressure and glucose levels, respectively. Following that, the short duration of the models could have an impact on the model's accuracy. As a result, it is prudent to utilize the model for short-term forecasting, as the mean of the series will remain constant for long-term forecasting. Additionally, our study examined only three of the district's 29 health facilities. As a result, the findings must be validated in other health facilities, cities, regions, or geographical locations to ensure accuracy. Finally, as it is a time series data evaluation with only one variable solely reliant on time, additional factors may contribute to the increase in hypertension and diabetes cases.

From this work, we recommend more advanced disease forecast models (such as the LSTM model) that can incorporate multiple factors to improve predicting precision and accuracy in the future. In addition, given the likelihood that the number of cases would increase, we further urge the local health directorate to strengthen context-specific and community-based intervention activities. Lifestyle modification should be a part of these programs, but it should not be the sole focus. Although underestimated, it is a critical component of preventing, controlling, and treating diabetes and hypertension. The changes include but are not limited to eating more fruits and vegetables, increasing physical activity (30 min of brisk walking per day), and reducing salt intake.

## 5. Conclusion

In this study, we used three models to predict the incidence of hypertension and diabetes in patients. The ARIMA time series model exhibited the highest accuracy among the tested models. The findings revealed a statistically significant suggestion for managerial and administrative decision-making practices.

## Figures and Tables

**Table 1:** ARIMA models comparison for further selection.

Models	AIC	RMSE	MAPE (%)	MAE	MSE
ARIMA (5, 2, 1)	337.75	37	11	31	789.67
ARIMA (5, 2, 4)	338.30	28	7	22	769.02
ARIMA (5, 2, 2)	337.89	43	19	39	799.75

Note: AIC: Akaike information criteria, RMSE: root mean square error, MAPE: mean absolute percentage error, MAE: mean absolute error, and MSE: mean square error.

**Table 2:** SARIMAX models comparison for further selection.

Models	AIC	RMSE	MAPE (%)	MAE	MSE
SARIMAX (1, 1, 1) × (1, 0, 1, 7)	298.82	79	19	66	5078
SARIMAX (1, 1, 1) × (1, 1, 0, 7)	288.65	68	16	55	4907
SARIMAX (1, 1, 1) × (1, 1, 1, 7)	282.50	67	14	56	4473

Note: AIC: Akaike information criteria, RMSE: root mean square error, MAPE: mean absolute percentage error, MAE: mean absolute error, and MSE: mean square error.

**Table 3:** LSTM models comparison for further selection.

Models	Optimizer	RMSE	MAPE (%)	MAE	MSE
1	Adam	36.00	8.60	27	1307
2	Adam	39.87	9.12	27.98	1895
3	Adam	37.99	9.36	27.72	1541

Note: Adam: adaptive moment estimation, RMSE: root mean square error, MAPE: mean absolute percentage error, MAE: mean absolute error, and MSE: mean square error.

**Table 4:** Forecasting performance of selected models.

Error metrics/models	ARIMA (5, 2, 4)	SARIMAX (1, 1, 1) × (1, 1, 1, 7)	LSTM
RMSE	28	67	36
MSE	769.02	4473	1307
MAE	22	56	27
MAPE (%)	7	14	8.6

Note: RMSE: root mean square error, MAPE: mean absolute percentage error, MAE: mean absolute error, and MSE: mean square error.

## Data Availability

We included all data analyzed during the study in this published article.
